# Occupational and leisure time physical activity in contrasting relation to ambulatory blood pressure

**DOI:** 10.1186/1471-2458-12-1002

**Published:** 2012-11-20

**Authors:** Els Clays, Dirk De Bacquer, Koen Van Herck, Guy De Backer, France Kittel, Andreas Holtermann

**Affiliations:** 1 Department of Public Health, Ghent University, University Hospital, (2) Block A, De Pintelaan 185, B-9000, Ghent, Belgium; 2 Department of Epidemiology and Health Promotion, School of Public Health, Université Libre de Bruxelles, Campus Erasmus CP 596, route de Lennik 808, B-1070, Brussels, Belgium; 3 National Research Centre for the Working Environment, Lersø Parkallé 105, DK-2100, Copenhagen, Denmark

**Keywords:** Physical activity, Occupational, Blood pressure

## Abstract

**Background:**

While moderate and vigorous leisure time physical activities are well documented to decrease the risk for cardiovascular disease, several studies have demonstrated an increased risk for cardiovascular disease in workers with high occupational activity. Research on the underlying causes to the contrasting effects of occupational and leisure time physical activity on cardiovascular health is lacking. The aim of this study was to examine the relation of objective and self-report measures of occupational and leisure time physical activity with 24-h ambulatory systolic blood pressure (BP).

**Methods:**

Results for self-reported physical activity are based on observations in 182 workers (60% male, mean age 51 years), while valid objective physical activity data were available in 151 participants. The usual level of physical activity was assessed by 5 items from the Job Content Questionnaire (high physical effort, lifting heavy loads, rapid physical activity, awkward body positions and awkward positions of head or arms at work) and one item asking about the general level of physical activity during non-working time. On a regular working day, participants wore an ambulatory BP monitor and an accelerometer physical activity monitor during 24 h. Associations were examined by means of Analysis of Covariance.

**Results:**

Workers with an overall high level of self-reported occupational physical activity as well as those who reported to often lift heavy loads at work had a higher mean systolic BP at work, at home and during sleep. However, no associations were observed between objectively measured occupational physical activity and BP. In contrast, those with objectively measured high proportion of moderate and vigorous leisure time physical activity had a significantly lower mean systolic BP during daytime, while no differences were observed according to self-reported level of leisure time physical activity.

**Conclusions:**

These findings suggest that workers reporting static occupational physical activities, unlike general physically demanding tasks characterized by dynamic movements of large muscle groups, are related to a higher daily systolic BP, while high objective levels of moderate and vigorous leisure time physical activity are related to lower daytime systolic BP. Ambulatory systolic BP may be a physiological explanatory factor for the contrasting effects of occupational and leisure time physical activity.

## Background

Moderate and vigorous leisure time physical activities are well documented to decrease the risk for cardiovascular disease (CVD) [1-3] and mortality [4,5]. In contrast, although much less research is available on the impact of occupational physical activity and findings are overall more inconsistent [6], several studies have demonstrated an increased risk for CVD and mortality in workers with high occupational physical activity [7-11]. Even in modern service economies, a sizeable fraction of the working population is exposed to high physical job demands [11,12], thus this is likely to infer a considerable public health impact.

Although some hypothetical physiological explanations to the increased risk for CVD from high occupational physical activity have been suggested, systematic epidemiological evidence on this topic is lacking [6]. Moreover, information about the underlying causes to the contrasting effects of occupational vs. leisure time physical activity on cardiovascular health would be particularly valuable for occupational and public health promotion [6]. Hence, research on the underlying causes to the contrasting effects of occupational and leisure time physical activity on cardiovascular health is particularly needed.

A potential explanatory physiological candidate to the contrasting effects of occupational and leisure time physical activity on cardiovascular health is blood pressure (BP) responses to physical activity. Elevated systolic BP is a well documented predictor for CVD and mortality [13]. Particularly daily ambulatory measured BP is shown as a strong predictor for cardiovascular events [14]. Physical activity plays a dominant role for the daily variations in ambulatory BP [15,16]. Leisure time physical activities like cycling and walking are shown to induce considerable reductions in ambulatory BP for up to 22 h following exercise [17,18]. Contrary, occupational physical activities like lifting, carrying and working with hands above shoulder level are known from laboratory studies to substantially elevate blood pressure [19]. Therefore, it is hypothesized that ambulatory systolic BP may be an explanatory factor for the contrasting relations of occupational and leisure time physical activity with cardiovascular health.

The aim of this study was to investigate the relation of both objective and self-report measures of occupational and leisure time physical activity with 24-h systolic BP in a sample of middle-aged workers from the Belgian Job Stress Project (BELTRESS).

## Methods

### Study population

BELSTRESS II was the second phase of an epidemiological cohort study on work-related stress and health and was conducted in 2002-’03 [20]. A total number of 2.821 men and women between 40 and 64 years old from nine companies or public administrations in Belgium volunteered to participate in the study by means of self-administered questionnaires and a bioclinical examination. The response rate of the study was 68.5%.

For a supplementary study with ambulatory measurements of BP and physical activity, four of the nine organizations were involved. This selection of organizations, which included two public administrations, one company from the secondary sector and one organization from the service sector, was based on practical reasons regarding feasibility of the fieldwork [21]. A subsample of participants was randomly selected within the four organizations. People taking medication for elevated BP were excluded from this study, as well as participants with a previous hospitalization for coronary heart disease. This study design was developed in order to obtain a population free from established CVD, in which relations can be investigated between work-related factors and BP patterns. The population of this study included 182 middle-aged workers (109 men and 73 women), which represented 66% of the invited subjects. Informed consent was obtained from all participants. Valid ambulatory measures of physical activity were available in 151 participants. The study was approved by the ethics committees of the University Hospital of Ghent and the Faculty of Medicine of the Free University of Brussels.

### Data collection

#### Questionnaire and anthropometric measurements

The questionnaire contained information on gender, age, educational level, occupational group and smoking status. Primary school level was defined as low education, secondary school level as medium education and high school or university as high education. Occupations were defined according to the International Standard Classification of Occupations and grouped into white-collar and blue-collar workers [22].

Participants were asked to indicate their usual level of leisure time physical activity on a set of four response possibilities: no weekly activity; only light physical activity during most weeks; heavy physical activity (i.e. resulting in sweating and elevated pulse rate) during 20 min or more once or twice per week; heavy physical activity during 20 min or more three times or more per week. High physical activity was attributed to persons who engage in heavy physical activity during 20 min or more at least three times per week, corresponding to the upper category.

The usual level of occupational physical activity was measured with a scale including five items from the Job Content Questionnaire [23]. The scale is composed of three items assessing physical exertion (high physical effort, lifting heavy loads, rapid physical activity) and two items assessing isometric loads (awkward body positions, awkward positions of head or arms), and has been shown an internationally validated tool to measure physical job demands [24]. The items were scored on a four-point Likert scale from ‘totally disagree’ to ‘totally agree’. For each of the five items, participants who (totally) agreed were assigned high levels of physical activity for that dimension. A summary scale of occupational physical activity was composed of the sum score of the five items and ranged from 5 to 20. This variable was highly skewed to the right and was therefore categorized. A minimum score of 13 was classified as a high level of overall occupational physical activity which generally corresponds to an exposure to three up to five of the individual items.

Stress at work was measured by items from the Job Content Questionnaire, based on the Job-Demand-Control model [23,24]. The psychological job demand scale was composed of the sum score of five items. Job control was composed of the sum score of two subscales: ‘skill discretion’ or the level of skill and creativity required on the job (six items) and ‘decision authority’ or the possibilities for workers to make decisions about their work (three items). Job strain was defined as the ratio of job demands over job control.

Participants were medically examined by trained members of the research team, following standardized procedures as described in a manual of operations. Height and body weight were assessed using calibrated devices while participants wore only light clothing and no shoes. Height was recorded to the nearest cm, body weight to the nearest 200 g. Body mass index (BMI) was calculated as body weight (in kg) divided by the square of the height (in m). Overweight was defined as a BMI of 25 or higher; those with a BMI of 30 or higher were classified as obese.

#### Ambulatory 24-h monitoring of blood pressure and physical activity

At the start of a regular working day, a trained member from the research team initiated the 24-h monitoring procedure at the workplace. For 24 h, participants wore an ambulatory BP monitor (Model 90121, SpaceLabs Medical, Inc., Redmond, WA, USA). The monitor was programmed to measure the arterial BP every half hour during the day (from 6 AM until 10 PM) and every hour at night (from 10 PM until 6 AM). During the day, every measurement was preceded by a warning tone. Participants were asked to keep their arm motionless and in a vertical position beside the body every time they heard the tone. In case a measurement failed due to excessive motion of the body, a new reading automatically followed a few minutes later. Before the automatic measurements started, two subsequent test readings were manually initiated to make participants familiar with the process.

In order to measure the physical activity during the 24-h monitoring, an activity monitor (Model 7164, Computer Science and Applications, Inc., Shalimar, FL, USA) was attached to the waist. This single-channel Actigraph continuously records accelerations of the body and has been proven a valid tool in assessing physical activity [25].

Moreover, participants were asked to perform their regular activities at work and at home during the monitoring period, and not to detach the devices until the next day. They were also asked to register their 24-h schedule (time spent at work, at home, and sleeping) in a diary.

Based on the information from the diaries, the average systolic ambulatory BP of every participant was calculated for the periods at work, at home, and during sleep. The average sleep BP could not be calculated for three participants because there were no valid readings at night. The average number of readings was 16 (± 3 s.d.) for the work period, 14 (± 3 s.d.) for the home readings, and 7 (± 2 s.d.) for the readings during sleep.

The activity monitor was programmed to register an activity count on every minute during the 24- h monitoring. Valid data could be processed in 151 participants; 31 were excluded due to technical software problems or because the device had been detached. The activity count data were processed separately for the time at work and leisure awake time. The average number of counts was 494 (± 75 s.d.) for the work period and 295 (± 205 s.d.) for the awake leisure time period. In order to assess the level of physical activity during work and leisure time in each participant, the proportion of work and leisure time spent in physical activity of moderate or vigorous intensity (≥ 2020 counts) was calculated for all participants. This cut-off was based on intensity thresholds for adults resulting from calibration studies that relate accelerometer counts to measured activity energy expenditure [26]. On group level, because this variable was highly skewed to the right, the upper quartile was classified as a high proportion of work and leisure time activity counts of moderate or vigorous intensity, as opposed to the lowest three quartile groups.

### Statistical analysis

Descriptive statistics were reported through numbers and proportions. Crude mean systolic ambulatory BP values were compared between occupational and leisure time physical activity groups, based on both questionnaire and monitoring data, with Independent Sample t-tests. Analyses of Covariance were used to assess multivariate associations adjusting for relevant confounders (gender, age, BMI, smoking, job strain and usual level of leisure time or occupational physical activity respectively). The use of linear regression analysis was not warranted since the physical activity variables were highly skewed and the condition of normally distributed residuals was not met.

All analyses were conducted using IBM SPSS Statistics software version 19 (IBM SPSS, Inc., Chicago, IL, USA).

## Results

Descriptive statistics are shown in Table 1. The sample included 40% women. About 58% of the participants were 50 years or older; mean age was 51.3 years (s.d. 4.6). Three quarters of them were white-collar workers; 38% had a low educational level. A high overall usual level of occupational physical activity was present in almost one fifth of the sample, while 13% reported to be highly physically active during leisure time.

**Table 1:** Sample characteristics for personal variables, socio-demographic factors and self-reported usual level of leisure time and occupational physical activity (N=182)

**Characteristics**	**N**	**%**
Gender		
Men	109	59.9
Women	73	40.1
Age		
< 45 years	16	8.8
45-49 years	60	33.0
50-54 years	62	34.1
≥ 55 years	44	24.2
Educational level		
Low	68	38.0
Medium	64	35.8
High	47	26.3
Job title		
Blue-collar	45	24.7
White-collar	135	74.2
Weight group		
Normal weight	70	38.5
Overweight	87	47.8
Obesity	25	13.7
Smoking		
Current regular smokers	42	23.1
Others	140	76.9
Leisure time physical activity		
Low	158	86.8
High	24	13.2
High physical effort at work		
High	46	25.3
Low	136	74.7
Lifting heavy loads at work		
High	33	18.1
Low	149	81.9
Rapid physical activity at work		
High	39	21.5
Low	142	78.5
Awkward body positions at work		
High	34	18.7
Low	148	81.3
Awkward positions of head or arms at work		
High	39	21.4
Low	143	78.6
Summary measure of occupational physical activity		
High	34	18.8
Low	147	81.2

Crude mean systolic ambulatory BP values were compared according to the usual level of occupational and leisure time physical activity based on questionnaire data (Table 2). Those with an overall high level of occupational physical activity as well as those often lifting heavy loads at work showed a 5 to 7 mmHg higher mean BP at work, at home and during sleep. A 4 to 5 mmHg higher mean work and sleep ambulatory BP was also observed in workers who often perform high physical effort and work with their head or arms in awkward positions. After adjusting for gender, age, BMI, smoking, job strain and usual level of leisure time physical activity, the relation between ambulatory BP and lifting heavy loads at work remained significant, while the association with the summary measure of occupational physical activity became borderline significant. No differences in systolic ambulatory BP were observed according to the self-reported level of physical activity during leisure time.

**Table 2:** Associations between self-reported occupational/leisure time physical activity and systolic ambulatory blood pressure (mmHg) (N=182)

**Occupational physical activity groups**	**crude mean SBP at work (SD)**	***P *(*T*-test)**	P* (Ancova)	**crude mean SBP at home (SD)**	***P *(*T*-test)**	P* (Ancova)	**crude mean SBP during sleep (SD)**	***P *(*T*-test)**	P* (Ancova)
Summary measure of occupational physical activity		**<0.01**	0.06		**<0.05**	0.11		**<0.01**	0.07
High	136.4 (13.8)			133.7 (12.3)			118.8 (13.7)		
Low	130.0 (11.0)			128.6 (10.1)			111.8 (10.7)		
High physical effort at work		**<0.05**	0.12		0.06	0.17		**<0.05**	0.10
High	134.4 (14.0)			132.2 (12.6)			116.8 (12.2)		
Low	130.2 (10.7)			128.7 (9.8)			112.0 (11.1)		
Lifting heavy loads at work		**<0.05**	**<0.05**		**<0.05**	**<0.05**		**<0.01**	**<0.05**
High	135.6 (14.6)			133.5 (12.6)			118.1 (13.1)		
Low	130.3 (10.8)			128.7 (10.0)			112.1 (11.0)		
Rapid physical activity at work		0.37	0.94		0.68	0.71		0.22	0.90
High	132.7 (13.3)			130.2 (11.2)			115.1 (12.5)		
Low	130.8 (11.4)			129.4 (10.5)			112.6 (11.3)		
Awkward body positions at work		0.07	0.67		0.24	0.79		**<0.05**	0.40
High	134.5 (12.3)			131.5 (12.6)			117.7 (13.7)		
Low	130.5 (11.5)			129.1 (10.1)			112.2 (10.8)		
Awkward positions of head or arms at work		**<0.01**	0.22		0.06	0.72		**<0.05**	0.48
High	135.7 (13.1)			132.4 (13.0)			117.4 (14.6)		
Low	130.0 (11.1)			128.8 (9.8)			112.1 (10.4)		
**Leisure time physical activity groups**	**crude mean SBP at work (SD)**	***P *(*T*-test)**	P** (Ancova)	**crude mean SBP at home (SD)**	***P *(*T*-test)**	P** (Ancova)	**crude mean SBP during sleep (SD)**	***P *(*T*-test)**	P** (Ancova)
	Leisure time physical activity		0.72	0.29		0.55	0.22		0.64	0.47
High	130.4 (11.5)			128.4 (11.1)			112.2 (8.1)			
Low	131.4 (11.8)			129.8 (10.6)			113.4 (12.0)			

Abbreviations: *SBP*, systolic blood pressure; *SD*, standard deviation; *Ancova*, Analysis of Covariance.

Significant associations at the 0.05 level are in bold.

* Adjusted for gender, age, BMI, smoking, job strain and usual level of leisure time physical activity.

** Adjusted for gender, age, BMI, smoking, job strain and usual level of occupational physical activity (summary measure).

Crude and adjusted mean systolic ambulatory BP values were compared according to objective measures of occupational physical activity (Table 3). No associations between occupational physical activity groups and systolic BP levels were observed.

**Table 3:** Associations between objective measures of occupational/leisure time physical activity and systolic ambulatory blood pressure (mmHg) (N=151)

**Occupational physical activity groups**	**crude mean SBP at work (SD)**	***P *(*T*-test)**	P* (Ancova)	**crude mean SBP at home (SD)**	***P *(*T*-test)**	P* (Ancova)	**crude mean SBP during sleep (SD)**	***P *(*T*-test)**	P* (Ancova)
Proportion of moderate or vigorous activity counts during working time		0.95	0.98		0.95	0.86		0.25	0.19
High (Q4)	130.7 (12.3)			129.3 (12.5)			114.8 (10.3)		
Low (Q1-Q3)	130.5 (11.6)			129.2 (10.1)			112.2 (12.1)		
**Leisure time physical activity groups**	**crude mean SBP at work (SD)**	***P *(*T*-test)**	P** (Ancova)	**crude mean SBP at home (SD)**	***P *(*T*-test)**	P** (Ancova)	**crude mean SBP during sleep (SD)**	***P *(*T*-test)**	P** (Ancova)
Proportion of moderate or vigorous activity counts during leisure time		**<0.01**	**<0.05**		**<0.01**	**<0.05**		0.42	0.71
High (Q4)	126.1 (10.2)			125.1 (10.4)			111.5 (9.7)		
Low (Q1-Q3)	132.0 (11.9)			130.5 (10.5)			113.3 (12.3)		

Abbreviations: *SBP*, systolic blood pressure; *SD*, standard deviation; *Ancova*, Analysis of Covariance; Q4, upper quartile: Q1-Q3, lowest 3 quartiles.

* Adjusted for gender, age, BMI, smoking, job strain and usual level of leisure time physical activity.

** Adjusted for gender, age, BMI, smoking, job strain and usual level of occupational physical activity (summary measure).

Crude mean systolic ambulatory BP values were compared according to objective measures of leisure time physical activity (Table 3). Workers with a high proportion of moderate or vigorous activity counts during leisure time showed a 5 to 6 mmHg significantly lower mean systolic BP level during the daytime (both at work and at home). After adjusting for gender, age, BMI, smoking, job strain and the usual level of occupational physical activity, the associations remained significant.

## Discussion

It has been convincingly argued that more research is needed to disentangle the roles of leisure time and occupational physical activity in affecting cardiovascular health [6,27]. Only few studies have simultaneously examined the differentiating effects of physical activities at work and during leisure time, often with conflicting results [28-31]. Moreover, research on the underlying physiological mechanisms of these contrasting effects is particularly lacking [6,27]. This study examined the relation of both objective and self-report measures of occupational and leisure time physical activity with 24-h ambulatory systolic BP in a sample of 182 workers. Main findings of this study were that workers with an overall high level of self-reported occupational physical activity as well as those who reported to often lift heavy loads at work had a higher mean systolic BP at work, at home and during sleep. However, no associations were observed between objectively measured occupational physical activity and BP. In contrast, those with objectively measured high proportion of moderate and vigorous leisure time physical activity had a significantly lower mean systolic BP during daytime, while no differences were observed according to self-reported level of leisure time physical activity.

Persons reporting high level of overall occupational physical activity and lifting heavy loads were observed to have a higher systolic ambulatory BP. This finding is in agreement with previous laboratory studies showing that occupational physically demanding work tasks like lifting, carrying and working with hands above shoulder level substantially enhance systolic BP [19]. However, persons with a high exposure to occupational physical activity and lifting of heavy loads did not only have an elevated systolic BP during work, but also at home and during sleep. Therefore, persons with these physical occupational exposures seem to have a generally increased level of systolic BP. Physical activity is well documented to influence systolic BP in the following 22 h [18]. However, it is unknown if the observed elevated systolic BP at home and during sleep among persons with high level of occupational physical activity and lifting of heavy loads are caused by these occupational physical exposures.

The ambulatory systolic blood pressure during work, home and sleep was more than 5 mmHg higher among those with high exposure of occupational physical activity and lifting of heavy loads compared to those with a low exposure. Elevated blood pressure is associated with CVD and mortality [13], and ambulatory systolic BP strongly predicts cardiovascular events [14]. Studies have shown reductions in systolic BP as small as 3 mmHg to decrease coronary heart disease by 5–9% and all-cause mortality by 4% in an average population [32,33]. Therefore, the finding of an elevated ambulatory systolic BP among people with high exposure of occupational physical activity and lifting of heavy loads provides a plausible physiological explanation for the increased risk for CVD and mortality among workers with high occupational physical activity [7-11]. However, prospective investigations of the potential mediating effect of ambulatory systolic BP on the relation between occupational physical activity and CVD are required before interpretations about causality can be drawn.

In contrast to the self-reported exposure to occupational physical activity, no significant associations between objectively measured occupational physical activity and ambulatory systolic BP were found. This finding may be explained by the specific characteristics of the applied objective monitoring system of physical activity. The single-channel Actigraph accelerometer is shown valid for measuring general level of physical activity [25], however it is not able to identify specific types of physical activity. Occupational physically demanding work tasks like lifting, carrying and working with hands above shoulder level are known from laboratory studies to enhance systolic BP [19], but occupational tasks characterized by dynamic movements of large muscle groups like walking are likely to reduce systolic BP [18]. Hence, our findings suggest that the higher systolic ambulatory BP in workers with high occupational physical activity is essentially due to exposure to more static or anaerobic physical activities at the workplace. Additional analyses in fact showed that self-report vs. objectively measured levels of occupational physical activity were only modestly correlated to each other (data not shown).

Persons with high objective levels of moderate and vigorous leisure time physical activity were found to have a lower level of systolic BP at work and home compared to persons with lower objective levels of leisure time physical activity. This finding is in accordance with previous studies showing acute reductions in systolic BP for up to 22 h after a bout of physical exercise and the general lower systolic BP among persons regularly performing leisure time physical activity [17,18]. Moreover, the finding of a reduced level of ambulatory systolic BP among persons with high levels of leisure time physical activity, but not among those with high levels of occupational physical activity fits well with the previously observed contrasting effects of leisure time and occupational physical activity on CVD and mortality [8]. Surprisingly though, a high level of self-reported physical activity during leisure time was not related to significantly lower systolic BP, which suggests that a broad 1-item assessment of leisure time physical activity with crude categorization of different levels might be insufficient to detect physiological response mechanisms.

### Methodological aspects

Participants in the BELSTRESS II study (N=2.821) were not recruited from a representative sample of the active working population in Belgium, which may limit the external validity of the results. Nonetheless, the study cohort covers a broad range of companies and occupational groups. For the supplementary study with ambulatory measurements of BP and physical activity, for which a much smaller sample size was aimed at, 182 participants were included, indicating a response rate of 66%. Valid ambulatory measures of physical activity were available in 151 persons. The presence of a severe selection bias is not likely since the reduced sample of 151 participants did not significantly differ from the total BELSTRESS II population in terms of general characteristics, self-reported occupational and leisure-time physical activity, or casual systolic BP at baseline. However, the 31 cases that did not provide valid accelerometer data showed a higher level of self-reported occupational physical activity compared to the 151 participants that did; hence the results for objective measures of physical activity at work might have been underestimated.

The major strength of this study is the use of objective 24-h ambulatory measurements of BP and physical activity. Ambulatory BP monitoring with 30 to 60 min measurements intervals has been shown to provide more reliable and accurate measures of BP than isolated clinic measures, and has the advantage that detailed BP indicators can be derived in terms of average work, home and sleep BP [34,35]. Concerning occupational and leisure time physical activity, both self-report and objective assessments have been used, which enables to gain more detailed insights in the relation with BP. The applied single-channel Actigraph accelerometer is shown valid for measuring general level of physical activity [25]. On the other hand, this monitoring system does not permit identifying the specific type of physical activity. Measurement of self-reported occupational physical activity was based on a standardized validated scale including five specific types of physical job demands [24]. Concerning the categorization of this variable, the cut-off of 13 on a scale ranging from 5 to 20 was based on logical grounds (high occupational physical activity corresponds to an exposure to three up to five of the individual items). However, since this categorization still is somewhat arbitrary, sensitivity analyses were performed with an alternative categorization using cut-off 12 (corresponding to an exposure to two up to five of the individual items, reported by 27% of the sample), which revealed a similar pattern of results. Due to the small sample size and hence limited statistical power, it was not feasible to use more detailed categorizations of physical activity to examine dose–response relations.

A weakness of the study is the single measurement of occupational and leisure time physical activity and ambulatory blood pressure. This increases the risk of misclassification which can be assumed to generally lead to an underestimation of the true associations in this population. Another limitation is the cross-sectional study design, not allowing inferences about causality. It is therefore of importance that the findings of this study are verified in prospective study designs. Although thorough adjustments were made for possible confounding factors in the multiple analysis, it remains possible that the observed findings are due to residual confounding. It could be for instance that people with different physical activity levels are in different ways attentive to their health which may affect their BP.

## Conclusions

Like previously shown in laboratory studies, workers with an overall high level of self-reported occupational physical activity as well as those reporting to often lift heavy loads at work were observed to have an increased systolic BP at work, at home and during sleep. These findings suggest that static occupational physical activities with the upper body or arms, unlike general occupational physically demanding tasks characterized by dynamic movements of large muscle groups, are related to a higher daily systolic BP. In contrast, those with objectively measured high proportion of moderate and vigorous leisure time physical activity had a significantly decreased systolic BP during daytime. These findings are in accordance with the observed contrasting effects of occupational and leisure time physical activity and CVD. Therefore, daily systolic BP is a plausible physiological mediator for the contrasting associations between occupational and leisure time physical activity and CVD. However, prospective studies of the potential mediating effects of ambulatory BP on the relation between occupational and leisure time physical activity and CVD are needed before causal interpretations can be drawn.

## Abbreviations

BP: Blood pressure; CVD: Cardiovascular disease.

## Competing interests

The authors declare that they have no competing interests.

## Authors’ contributions

EC, DDB, GDB and FK were involved in the conception, design and data collection of the BELSTRESS II study. EC carried out the analysis of the data. EC and AH interpreted the data and drafted the manuscript. DDB, KVH, GDB and FK critically revised the manuscript and helped interpreting the data. All authors have given final approval of the version to be published.

## Pre-publication history

The pre-publication history for this paper can be accessed here:

http://www.biomedcentral.com/1471-2458/12/1002/prepub
